# A Method for Assessing the Prevalence of Food Insecurity in Older Adults Based on Resource Constraints and Food-Related Physical Functioning Limitations

**DOI:** 10.1016/j.cdnut.2024.104494

**Published:** 2024-10-29

**Authors:** AnnieBelle J Sassine, Matthew P Rabbitt, Alisha Coleman-Jensen, Alanna J Moshfegh, Nadine R Sahyoun

**Affiliations:** 1 Department of Nutrition and Food Science, University of Maryland, College Park, MD, United States; 2 Economic Research Service, U.S. Department of Agriculture, Washington, DC, United States; 3 Beltsville Human Nutrition Research Center, Agricultural Research Service, U.S. Department of Agriculture, Beltsville, MD, United States

**Keywords:** food security, physical functioning limitations, older adults, NHANES, depression, self-reported health, cross-classification method, Healthy Eating Index-2015

## Abstract

**Background:**

Older adults with food-related physical functioning limitations often face food insecurity because of challenges that go beyond resource constraints. Difficulties with food acquisition at retailers, and food preparation and consumption are not captured by the United States Department of Agriculture’s food security measure.

**Objectives:**

The objective of this study was to develop a method to assess the overall prevalence of food insecurity regardless of underlying cause using validated measures that capture both food-related physical functioning limitations and resource-constraint food hardships. It also aims to examine the validity of this method in relation to sociodemographic characteristics, health, appetite, and dietary outcomes.

**Methods:**

Using information from National Health and Nutrition Examination Surveys (2007–2018), 10,237 adults aged 60 y and older with complete food security and food-related physical functioning limitations data were included in the study. Comparisons of the cross-classification method and the standard food security methods are made and their relationships with depression, self-reported health, and healthy eating index 2015 are examined using multiple linear regression models.

**Results:**

Using the cross-classification method, prevalence of low and very low food security because of resource constraints and food-related physical functioning limitations was found to be higher (13.6% and 7.3%, respectively) compared with food insecurity based solely on resource constraints (4.7% and 3.3%, respectively) or food-related physical functioning limitations (11.4% and 4.4%). Low and very low food security levels using the cross-classification method were associated with higher odds of depression and poor self-reported health, compared with the standard United States Department of Agriculture (USDA) resource-constrained food security measure.

**Conclusions:**

The cross-classification method enables the identification of potentially food insecure older adults who might be classified otherwise if only each measure is used alone. This method serves as guidance for using both measures jointly to estimate food insecurity, regardless of its cause.

## Introduction

Food security is defined by the United Nations as “when all people, at all times, have physical, social, and economic access to sufficient, safe, and nutritious food that meets their dietary needs and food preferences for an active and healthy life” [1]. This definition, which evolved over time, underlines the multidimensional aspect of food insecurity. However, current survey modules assessing food insecurity primarily focus on financial or economic hardships related to food. The decision to focus on the central “resource-constraint” dimension of food insecurity in the United States Department of Agriculture’s (USDA) Household Food Security Survey Module was made during the First National Conference on Food Security Measurement and Research [2]. Although the lack of resources for food is a common constraint, food insecurity can also stem from physical inability to access food because of difficulty in shopping and cooking, and may also encompass the “utilization” dimension that involves difficulty in consuming food [3]. This is particularly relevant as the number of older adults is growing with consequently higher prevalence of physical limitations because of chronic health conditions and other limitations of older age, that may hinder food access and lead to food insecurity [4]. A study by Jackson et al. [5] showed that older adults with 4 or more physical functioning limitations were almost 3 times more likely to report very low food security (VLFS) than older adults without physical functioning limitations. Petersen et al. [6] also showed that functional limitations among older adults were significantly associated with more severe food insecurity. The “State of Senior Hunger in America” annual report also highlighted that food insecurity is more common among seniors living in households where a member has a disability, compared with those households without a disability [7].

A recent study by Vaudin et al. [8] showed that older adults with food-related physical functional limitations and who lived in food insecure households because of resource constraints had the highest mean depression score and lowest healthy eating index 2015 (HEI-2015) score in comparison to food secure older adults, those with economic food insecurity alone, and those with physical functioning related food insecurity alone. There is also substantial evidence showing that functional limitations are associated with higher levels of depressive symptoms in older adults [[9], [10], [11]]. In fact, 1 study by Soriano et al. [12] found that older adults who had difficulties preparing meals reported more severe depressive symptoms. This is particularly alarming as depressive disorders are the most common psychiatric conditions among older adults [13,14] and they are a major risk factor for disability and mortality [15].

A scale based on food-related physical functioning limitations was recently developed and validated using data from the National Health and Nutrition Examination Survey (NHANES) 2013–2018 cycles focusing on adults aged 60 y and older. The scale which is composed of 6 questions demonstrated internal consistency and construct validity, evidenced by significant associations with self-reported health (SRH) status, self-reported diet quality, and food insecurity [16]. The intent of the food-related physical functioning food security tool is to complement the USDA food security tool in evaluating food hardship among older adults by capturing an additional dimension of food security. In light of this, the present study introduces a novel cross-classification approach to assess and report on the prevalence of food insecurity among older adults that accounts for food security related to both financial constraints and food-related physical functioning limitations. This method also aims to test whether the newly proposed food security cross-classification system is consistent with sociodemographic indicators, SRH, depression, and diet quality using self-reported appetite and the HEI-2015 among older adults participating in the NHANES 2007–2018 cycles.

The proposed cross-classification method can provide a simplified approach for estimating the prevalence of food insecurity because of resource constraints and food-related physical functioning limitations among older adults, while also incorporating severity levels of food insecurity.

## Methods

### Survey design and study population

NHANES is a series of cross-sectional surveys using a complex multistage probability design to collect data from a representative sample of the civilian, noninstitutionalized United States population. It has been fielded annually and data are released every 2 y. The survey combines home interviews and physical examinations in Mobile Examination Centers (MECs) to assess the health and nutritional status of adults and children [17]. NHANES over-samples persons 60 y and older, as well as African Americans and Hispanics, to produce reliable statistics [17]. An informed consent was obtained from all participants. NHANES is approved by a Statistics Research Ethics Review Board.

This study’s population consisted of adults 60 y of age and older who participated in 6 NHANES cycles (2007–2008, 2009–2010, 2011–2012, 2013–2014, 2015–2016, and 2017–2018). This study was a secondary analysis of a publicly available and de-identified dataset and, therefore, was deemed exempt from further institutional review under federal regulation 45 CFR 46.101(b).

### Food security measures

#### United States Adult Food Security Survey Module

Food security from the adult perspective was measured using the first 10 items of the USDA Household Food Security Survey Module [commonly referred to at the Adult Food Security Survey Module (AFSSM)], a widely used instrument, which includes questions on the experiences and behaviors related to insufficient resources to acquire food over the past 12 mo. These questions were part of the Food Security section in the NHANES survey and were administered in participants’ homes. High food security (HFS) is defined as 0 affirmative responses, marginal food security (MFS) as 1–2 affirmative responses, low food security (LFS) as 3–5 affirmative responses, and VLFS as 6–10 affirmative responses [18]. Throughout the article, the AFSSM is referred to as “resource-constraint” food security.

#### Food-related physical functioning limitations

Food insecurity because of physical functioning limitations related to food acquisitions and consumption was assessed using the previously developed and validated Physical Food Security instrument (PFS) [16]. PFS consists of 6 questions related to difficulty performing the following tasks: standing for ∼2 h, lifting or carrying heavy objects, going out shopping, reaching up over head, preparing own meals, and using a fork or knife to eat. These questions are found in the Physical Function Questionnaire section of NHANES under physical limitations and are distinct questions that capture the unique dimension of food-related physical functioning limitations. Questions regarding physical functioning were also assessed during in-home interviews with participants. Participants who answered having “some difficulty,” “much difficulty,” or “unable to do the activity” to the questions had an affirmative response and those who answered “no difficulty performing this activity” had a negative response. Those with missing responses to any of the 6 items were considered missing (*n* = 1459).

The PFS instrument is categorized as follows: High food-related physical functioning security with zero affirmative responses to food-related physical functioning limitations (H-PFS), marginal food-related physical functioning security with 1–2 food-related physical functioning limitations (M-PFS), low food-related physical functioning security with 3–4 food-related physical functioning limitations (L-PFS), and very low food-related physical functioning security with 5–6 affirmative responses to the food-related physical functioning limitations (VL-PFS).

#### Cross-classification of resource-constraint food insecurity and food-related physical functioning limitations

In light of the multidimensional nature of food insecurity, the purpose of the cross-classification approach is to estimate the prevalence of food insecurity by integrating factors related to resource constraints and food-related physical functioning limitations. To determine whether the 2 measures could be combined into a single measure, we performed an exploratory factor analysis on all 16 items from the 6-item PFS and the 10-item AFSSM. The analysis revealed a 3-factor structure. The first factor, associated with “resource-constraint” food insecurity, had an eigenvalue of 5.67. The second factor, related to food-related physical functioning limitations, had an eigenvalue of 2.80. The third factor, which consisted of only 2 items – not eating for a whole day and the frequency of such occurrence, was indicative of the severity/frequency of food insecurity, with an eigenvalue of 1.58. The results underscore the multidimensionality of food security measures, suggesting that items from both scales cannot be merged into a single, unidimensional tool. In addition, a single unified scale that sums the raw scores from both measures may not accurately identify the severity level of food insecurity. For instance, a score ≥10 would signify VLFS. However, if an older adult had a score of 6 of 16 pertaining only to physical limitations, they would not be classified as having VLFS according to the additive measure. Alternatively, reporting the results of the 2 measures separately may result in double counting food insecure individuals and not provide a single severity measure.

As a result of our exploratory work, we suggest a novel cross-classification method that categorizes overall food insecurity for older adults or adults with food-related physical functioning limitations on the basis of the combination of the results of each scale, and their severity levels. The cross-classification method proposed in this article builds upon the method developed by Nord and Coleman-Jensen [19] for assigning a food security status to households with children while capturing the multidimensional measure related to adult and child food hardship.

According to the proposed cross-classification method, older adults who have zero raw scores on both scales are classified as having high food security (HFS/H-PFS). Those considered marginally food secure because of responses to 1 or both instruments are considered as having cross-classified marginal food security (MFS/M-PFS) and adults considered low food secure because of responses to 1 or both instruments are considered as having cross-classified low food security (LFS/L-PFS). Finally, adults considered very low food secure because of responses to 1 or both instruments are considered as having cross-classified very low food security (VLFS/VL-PFS) (Table 1) [16]. In summary, as shown in Table 1, the primary goal of the cross-classification approach is to more accurately represent the multidimensional definition of food insecurity whereby a participant cannot be considered food secure if they are insecure in any 1 aspect of the definition. It is therefore referred to as the multidimensional cross-classification approach. The frequencies for each subcategory of the cross-classification method are summarized in Supplemental Table 1.

**Table 1:** Moderator Guides Used for Listening Sessions and Pre-/Post Pilot Grantee Interviews

	Resource constraint food security (AFSSM)^1^			
*Food-related physical functioning limitations*^2^	**High food security (HFS)** **(Raw score 0 on AFSSM)**	**Marginal food security (MFS)** **(Raw scores 1-2 on AFSSM)**	**Low food security (LFS)** **(Raw scores 3-5 on AFSSM)**	**Very low food security (VLFS)** **(Raw scores 6-10 on AFSSM)**
**High food-related physical functioning security (H-PFS)** **(Raw score 0 on PFS)**	HFS/H-PFS	MFS/M-PFS	LFS/L-PFS	VLFS/VL-PFS
**Marginal food-related physical functioning security (M-PFS)** **(Raw scores 1-2 on PFS)**	MFS/M-PFS	MFS/M-PFS	LFS/L-PFS	VLFS/VL-PFS
**Low food-related physical functioning security (L-PFS)** **(Raw scores 3-4 on PFS)**	LFS/L-PFS	LFS/L-PFS	LFS/L-PFS	VLFS/VL-PFS
**Very low food-related physical functioning security (VL-PFS)** **(Raw scores 5-6 on PFS)**	VLFS/VL-PFS	VLFS/VL-PFS	VLFS/VL-PFS	VLFS/VL-PFS

Abbreviations: HFS, high food security; H-PFS, high food-related physical functioning security; LFS, Low food security; L-PFS, Low food-related physical functioning security; MES, marginal food security; M-PFS, marginal food-related physical functioning security; VLFS, Very low food security; VL-PFS, Very low food-related physical functioning security.

1 Resource-constraint food security was measured using USDA Adult Food Security Survey Module (AFSSM).

2 Food-related physical limitations were evaluated using the 6-item Physical Food Security tool, which was developed previously [16].

### Sociodemographic variables

Self-reported sociodemographic variables included age (years; categorized as 60–69, 70 y and older), sex (male or female), race and ethnicity (Mexican American, Other Hispanic, non-Hispanic white, non-Hispanic Black, other non-Hispanic race), and education (less than high school, high school graduate, greater than high school graduate). Education level data were considered missing for 16 observations where participants either refused to answer (*n* = 3) or did not know their educational attainment (*n* = 13). The income-to-poverty ratio was categorized as ≤1.85 to represent participants who are at or below 185% of the poverty threshold. Because of NHANES not computing values for incomes reported as <$20,000 or ≥$20,000, there were 932 missing observations in the analysis examining the association between income-to-poverty ratio and the cross-classified scale. Marital status was dichotomized into 2 categories, married or living with partner compared with widowed, divorced, separated, or never married. Marital status data were considered missing for 8 participants: 6 who refused to answer and 2 who did not know their status. The total number of people in the household was also included and categorized as 1, 2 or 3 and more. All sociodemographic data were collected from the demographic’s questionnaire of the NHANES survey, which was administered in participants’ homes.

### Dependent variables

The purpose of exploring the associations between dependent variables and the cross-classified food security scale is to serve as support and validation for the cross-classification method.

#### Self-reported medical conditions

A variable for significant weight loss was used because of its association with depression and medical conditions [20]. An individual was considered to have significant weight loss if he/she reported losing ≥10% of body weight from their original weight within the past year [21,22]. Information on weight loss was collected from the Weight History questionnaire of NHANES, which was administered in the home. Chronic diseases have been associated with both food insecurity and physical functioning limitations [[23], [24], [25]]. Consequently, self-reported medical conditions were also examined in relation to food security status and included arthritis, coronary artery disease, stroke, cancer, and diabetes, on the basis of the question “Has a doctor or other health professional ever told {you/sample person (SP)} that {you/s/he} …had (insert medical condition)?” This question was assessed in the medical conditions section of NHANES, which was administered in participants’ homes. Participants who responded “don’t know” to any of the listed medical conditions or refused to answer were excluded from the specific analysis examining the relationship between each medical condition and the cross-classified scale. Consequently, 29 observations were omitted from the arthritis bivariate analysis, 66 from coronary artery disease, 10 from cancer, 18 from stroke, and 4 from diabetes.

#### Depression screening score

The depression score was derived from the NHANES Patient Health Questionnaire (PHQ-9), a valid and reliable 9-item screening instrument, administered in the MEC. The questionnaire includes questions about the frequency of symptoms of depression over the past 2 wk. Respondents who answered “not at all” were given a score of 0, indicating a negative response, and those who answered “several days,” “more than half the days,” or “nearly every day” were given a score corresponding to 1, 2, and 3, respectively. A total score ranging from 0 to 27 was calculated. Older adults who did not participate in the clinical examination were also excluded from the analysis (*n* = 411). Those who refused to answer or had a missing response to any of the 9 items were excluded (*n* = 1206), resulting in a total sample size of 9031. Scores of ≥10 were used to denote the presence of depression [26,27].

#### Appetite

The question on poor appetite or overeating, which is included in the PHQ-9, was assessed in relation to the cross-classified food security status. Additionally, a separate question on whether problems related to depression have made it difficult for the participant to do his/her work, take care of things at home or get along with people was also examined in relation to cross-classified food security status. The variable was dichotomized into “yes” for responses corresponding to “somewhat difficult,” “very difficult,” or “extremely difficult” and “no” for a response of “not at all difficult.” There were 738 missing responses, resulting in a sample size of 9088 for the appetite variable.

#### Self-reported health

SRH status collected during a household interview of the participant was examined in association with the food security and physical functioning limitation variables. This is a reliable quick assessment of one’s subjective health using a single question, “Would you say {your/SP’s} health in general is excellent, very good, good, fair or poor?” [28] For this study, the responses were dichotomized as excellent/very good/good and fair/poor in bivariate analysis, on the basis of previous studies that used the same classification approach [29,30]. In the logistic regression model, the SRH status was used as a dichotomous variable with poor/fair indicating “poor SRH” and the referent category was “excellent/very good/good” SRH status.

#### Healthy Eating Index

The dietary interview component of NHANES is referred to as “What We Eat in America” (WWEIA) [31]. Trained interviewers, proficient in both English and Spanish, collect two 24-h dietary recalls. The first recall occurs in-person in a private room at the MEC, whereas the second is conducted by telephone 3–10 d later. These recalls follow the Five-step USDA Automated Multiple-Pass Method, with standard measuring guides assisting participants in estimating portion sizes [32]. For assessing diet quality, we calculated the Healthy Eating Index (HEI-2015) score using the National Cancer Institute’s Per-Person Simple HEI Scoring Algorithm, on the basis of NHANES (2007–2018) data from two 24-h dietary recalls [33]. Participants who did not have complete or reliable dietary recalls on day 1 (*n* = 44) or day 2 (*n* = 69), as well as those who did not undergo the dietary recall on day 1 (*n* = 635) and day 2 (*n* = 915) were excluded from the analysis. This resulted in a sample of 8163 older adults with complete dietary recalls. Total calorie data were missing for 6 participants, preventing the computation of their HEI scores. Consequently, the final sample size was 8157 older adults.

The HEI-2015 evaluates the quality of the diet on the basis of its adherence to the 2015–2020 Dietary Guidelines for Americans (DGA) [34]. It consists of 13 components in total, each contributing to a cumulative score that ranges from 0 to 100. According to the simple HEI scoring method, first the ratio of the dietary constituent to energy is constructed, which is then scored on the basis of the scoring standards. The total score is based on the sum of component scores and its mean represents the total scores across individuals. A higher score indicates a greater intake of beneficial food components such as total vegetables, greens and beans, total fruit, whole fruit, whole grains, dairy, total protein, seafood and plant protein, and a favorable ratio of fatty acids. A higher score also indicates a lower consumption of foods recommended for limited intake, such as sodium, refined grains, saturated fat, and added sugars [33].

### Statistical analysis

All data were imported into Stata/BE 17.0 (TX) from the NHANES database found on the Centers for Disease Control and Prevention (CDC) website. Data for the 6 NHANES cycles were pooled and the data of adults 60 y and older were analyzed.

To ascertain whether all the assumptions for factor analysis were met and if there were adequate intercorrelations to conduct the factor analysis, we utilized 2 indicators: Barlett’s test of sphericity and the Kaiser-Meyer-Olkin (KMO) measure of sampling adequacy. For the selected 16 items, Barlett’s test was significant (*P* < 0.0001), and the KMO value was 0.85, surpassing the recommended value of 0.5. As a result, factor analysis was carried out on the combined scale.

The multiyear sampling weights for the combined 12-y (12 y) cycles from 2007 to 2018 NHANES were constructed by dividing the 2-y sample weights by the number of 2-y cycles, in this case 6 cycles [35].

The selection of sampling weights depended on the type of analysis conducted. For analyses containing only variables collected in the in-home interview, like the distribution of sociodemographic characteristics across food security status, the sampling interview weight “wtint12yr” was used. For analyses containing some variables collected in the MEC, such as the depression and SRH, the sampling MEC weight “wtmec12yr” was used. For analysis looking at HEI-2015 scores, the sampling 2-d dietary weight "wtdr2d12yr” was applied to account for survey data.

When looking at the distribution of sociodemographic and medical conditions data by food security status, Rao-Scott weighted chi-square tests were used. When testing the association between the cross-classified food security status and depression or HEI-2015 total scores, we first conducted independent *t*-tests to test for differences in the mean depression and total HEI-2015 score across food security categories. The resulting *P* value was multiplied by 6 to adjust for multiple comparisons. Second, we conducted unadjusted and adjusted logistic regressions to look at the associations between food security levels and depression and SRH as dichotomous outcomes.

We also used logistic and linear regression models to examine the associations between the food security measures and depression, SRH, and HEI-2015. All regression analyses were weighted and adjusted for age, sex, race, educational level, and marital status. The linear regression models were then compared using the seemingly unrelated estimation (*suest*) command in Stata version 17.0. The purpose of this comparison was to assess whether differences in the odds ratios (OR) or regression coefficients existed between the standard USDA food security tool and the cross-classification method.

## Results

**FIGURE 1:**
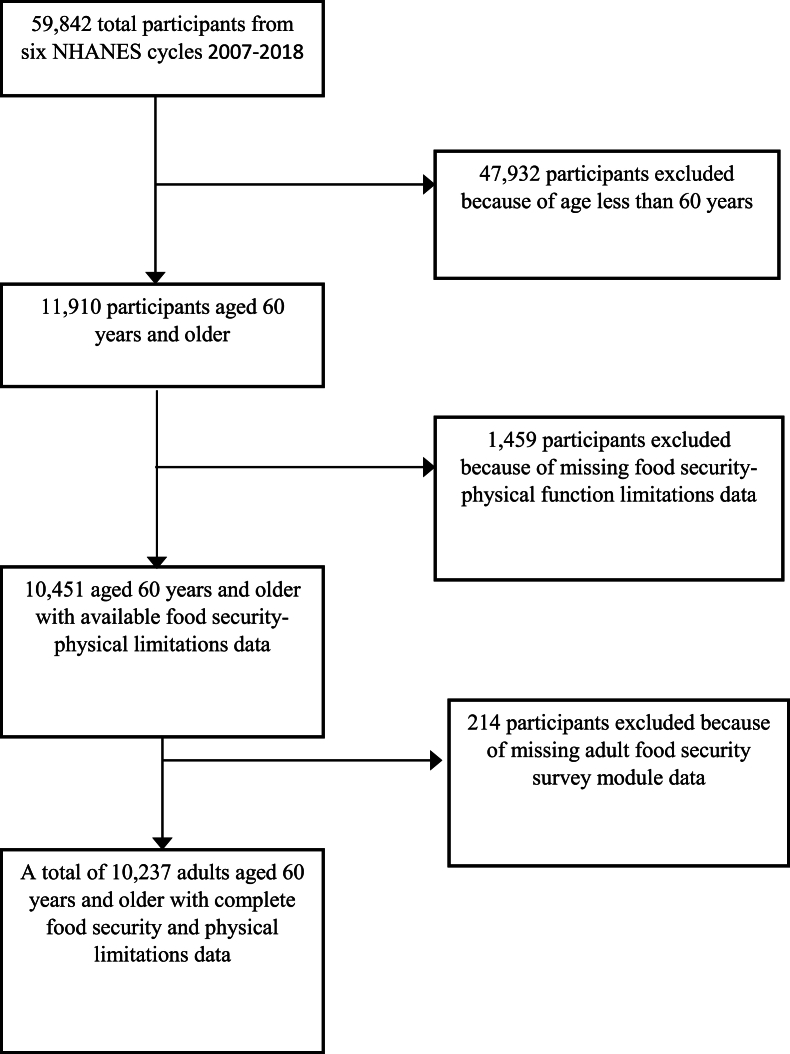
Sample selection flowchart from National Health and Nutrition Examination Survey (NHANES 2007–2018).

Sample selection is described in Figure 1. Out of 59,842 total participants, only adults who were aged 60 y and older and had complete data on food security and food-related physical functioning limitations were included in the analysis, resulting in a final sample of 10,237 participants (Figure 1). Table 2 shows the prevalence of food security levels according to 3 food security classification approaches: the multidimensional cross-classification method, the resource-constraint food security using USDA’s 10-item AFSSM, and the food-related physical functioning food security using the 6-item PFS scale. When using the cross-classification approach, the prevalences of low and VLFS were 13.6%, and 7.3%, respectively. These prevalences were higher compared with those based on the resource-constraint food security tool (4.7% and 3.3%) and the food-related physical functioning limitations tool (11.4% and 4.4%). Consequently, HFS, as determined by the multidimensional cross-classification method, was lower among our older adult population (Table 2). This is because participants had to be categorized as high on both the economic constraints and food-related physical limitation dimensions of the scale to be cross-classified as “high food security” in our proposed method.

**TABLE 2:** Prevalence of food security among adults ages 60 y and older by different food security measures, NHANES 2007–2018.

	Food security classification according to resource constraints and physical functioning limitation (*n* = 10,237)				Resource-constraint food security using USDA AFSSM (*n* = 10,237)				Food-related physical functioning limitations using PFS tool (*n* = 10,237)			
	High	Marginal	Low	Very low	High	Marginal	Low	Very low	High	Marginal	Low	Very low
Frequency (*n*)	4285	3065	1815	1072	7914	947	829	547	5092	3134	1403	608
Weighted proportion (%)	48.3	30.8	13.6	7.3	86.0	6.0	4.7	3.3	52.7	31.4	11.4	4.4

AFSSM refers to USDA Adult Food Security Survey Module found in NHANES; PFS refers to the six-item Physical Food Security tool.

On the basis of the cross-classification method, participants in the cross-classified low and VLFS categories were more likely to be females, aged 70 y and older, Hispanic, or Black than those in HFS category (Table 3). They were also more likely to be widowed, separated, divorced, or never married with a lower educational attainment, all factors commonly associated with an increased risk for food insecurity because of resource constraints. The percentage of older adults with incomes less than or equal to 185% of the federal poverty level was higher among those at higher risk of low and VLFS under the cross-classification method. Older adults living alone or living with 3 or more persons were more likely to be low or very low food secure than older adults living with 1 other person (Table 3).

**TABLE 3:** Distribution of sociodemographic characteristics across cross-classified food security status in adults aged 60 y and older, NHANES 2007–2018.

	*n*	Food security classification according to resource constraints and food-related physical functional limitations				*P* value
		High food security (HFS/H-PFS) (= 4285)	Marginal food security (MFS/M-PFS) (*n* = 3065)	Low food security (LFS/L-PFS) (*n* = 1815)	Very low food security (VLFS/VL-PFS) (*n* = 1072)	
10,237 sociodemographic characteristics						
Sex (%)	10,237					
Male		50.0	41.7	33.5	40.7	<0.0001
Female		50.1	58.3	66.5	59.3	
Age group (y) (%)	10,237					
60–70		66.4	52.4	52.4	56.0	<0.0001
70+		33.6	47.7	47.6	44.0	
Ethnicity (%)	10,237					
Mexican American		2.86	3.45	6.47	8.32	<0.0001
Other Hispanic		2.63	2.93	5.98	7.70	
Non-Hispanic White		81.8	80.6	66.3	62.2	
Non-Hispanic Black		7.08	7.97	13.5	13.8	
Other - Including multiracial		5.60	5.06	7.76	7.99	
Marital status (%)	10,229					
Married or living with partner		71.1	62.4	52.1	46.1	<0.0001
Widowed, divorced, separated, or never married		28.9	37.6	47.9	54.0	
Total number of people in the household (%)	10,237					
1		20.8	24.9	29.3	27.8	<0.0001
2		60.6	57.0	43.8	40.0	
3+		18.6	18.1	26.9	32.2	
Educational level (%)	10,221					
<High school		11.6	17.3	29.2	33.6	<0.0001
High school		22.9	26.4	27.4	25.4	
>High school		65.6	56.2	43.3	41.0	
Income-to-poverty ratio (%)^1^	9305					
≤1.85		17.6	29.7	50.3	64.1	<0.0001
>1.85		82.4	70.3	49.7	35.9	

Abbreviations: HFS, high food security; H-PFS, high food-related physical functioning security; LFS, Low food security; L-PFS, Low food-related physical functioning security; MES, marginal food security; M-PFS, marginal food-related physical functioning security; VLFS, Very low food security; VL-PFS, Very low food-related physical functioning security.

^1^Income to Poverty Ratio determined by ratio of family income to national poverty threshold for the household. HFS/H-PFS refers to cross-classified high food security; MFS/M-PFS refers to cross-classified marginal food security; LFS/L-PFS refers to cross-classified low food security, and VLFS/VL-PFS refers to cross-classified very low food security.

Additionally, there were significant differences in the prevalence of self-reported arthritis, diabetes, and stroke across cross-classified food security categories (*P* < 0.0001) (Table 4). Cancer, however, was highest among older adults with a cross-classified MFS status. Significant weight loss was also more likely to be reported among those with more severe levels of cross-classified food insecurity, compared with those in the HFS category (Table 4). Also, those with cross-classified VLFS were almost 4 times more likely to report having poor appetite or overeating and 6 times more likely to report difficulties doing work, taking care of things at home, or getting along with people as compared with older adults with HFS (Table 4). Table 4 also shows that older adults with cross-classified HFS had a significantly higher mean HEI-2015 total score (58.5 ± 0.4) compared with cross-classified marginal (56.5 ± 0.4), low (55.1 ± 0.6), and very low (53.4 ± 0.8) food secure older adults.

**TABLE 4:** Self-reported medical conditions, health-related characteristics, and HEI-2015 scores across cross-classified food security categories because of resource constraints and food-related physical functioning limitations, in adults aged 60 y and older, NHANES 2007–2018.

	*n*	Food security classification according to resource constraints and food-related physical functional limitations				*P* value
		High food security (HFS/H-PFS) (*n* = 4285)	Marginal food security (MFS/M-PFS) (*n* = 3065)	Low food security (LFS/L-PFS) (*n* = 1815)	Very low food security (VLFS/VL-PFS) (*n* = 1072)	
Self-reported medical conditions						
Arthritis (%)	10,208					
Yes		38.5	61.2	66.6	68.2	<0.0001
No		61.5	38.8	33.4	31.8	
Coronary artery disease (%)	10,170					
Yes		7.59	11.2	10.4	16.0	<0.0001
No		92.4	88.8	89.7	84.1	
Diabetes, or borderline diabetes (%)	10,233					
Yes		18.3	24.0	32.8	37.3	<0.0001
No		81.7	76.0	67.2	62.8	
Stroke (%)	10,219					
Yes		2.88	6.81	12.5	19.4	<0.0001
No		97.1	93.2	87.5	80.6	
Cancer (%)	10,227					
Yes		23.0	27.6	25.7	22.0	0.002
No		77.0	72.4	74.3	78.0	
Other characteristics						
Significant weight loss^1^ (%)	9957					
Yes		5.5	8.6	12.5	14.8	<0.0001
No		94.5	91.4	87.5	85.2	
Depression-related difficulty doing work, taking care of things at home or getting along with people (%)	9058					
None		93.1	85.0	73.7	60.4	<0.0001
Any difficulty		6.95	15.0	26.3	39.6	
Poor appetite or overeating (%)	9088					
Yes		11.1	20.6	31.9	39.7	<0.0001
No		88.9	79.4	68.1	60.3	
Healthy eating index (HEI-2015) total score, mean, 95% CI	8157	58.5^2^ (57.7, 59.4)	56.5 (55.6, 57.3)	55.1 (54.0, 56.3)	53.4 (51.7, 55.1)	

Abbreviations: HFS, high food security; H-PFS, high food-related physical functioning security; LFS, Low food security; L-PFS, Low food-related physical functioning security; MES, marginal food security; M-PFS, marginal food-related physical functioning security; VLFS, Very low food security; VL-PFS, Very low food-related physical functioning security.

HFS/H-PFS refers to cross-classified high food security; MFS/M-PFS refers to cross-classified marginal food security; LFS/L-PFS refers to cross-classified low food security, and VLFS/VL-PFS refers to cross-classified very low food security.

1 Participants who lost ≥10% of body weight from their original weight 1 y ago were classified as having significant weight loss.

2 Significantly different than all other categories after adjusting for multiple comparisons (*P* < 0.01).

**FIGURE 2:**
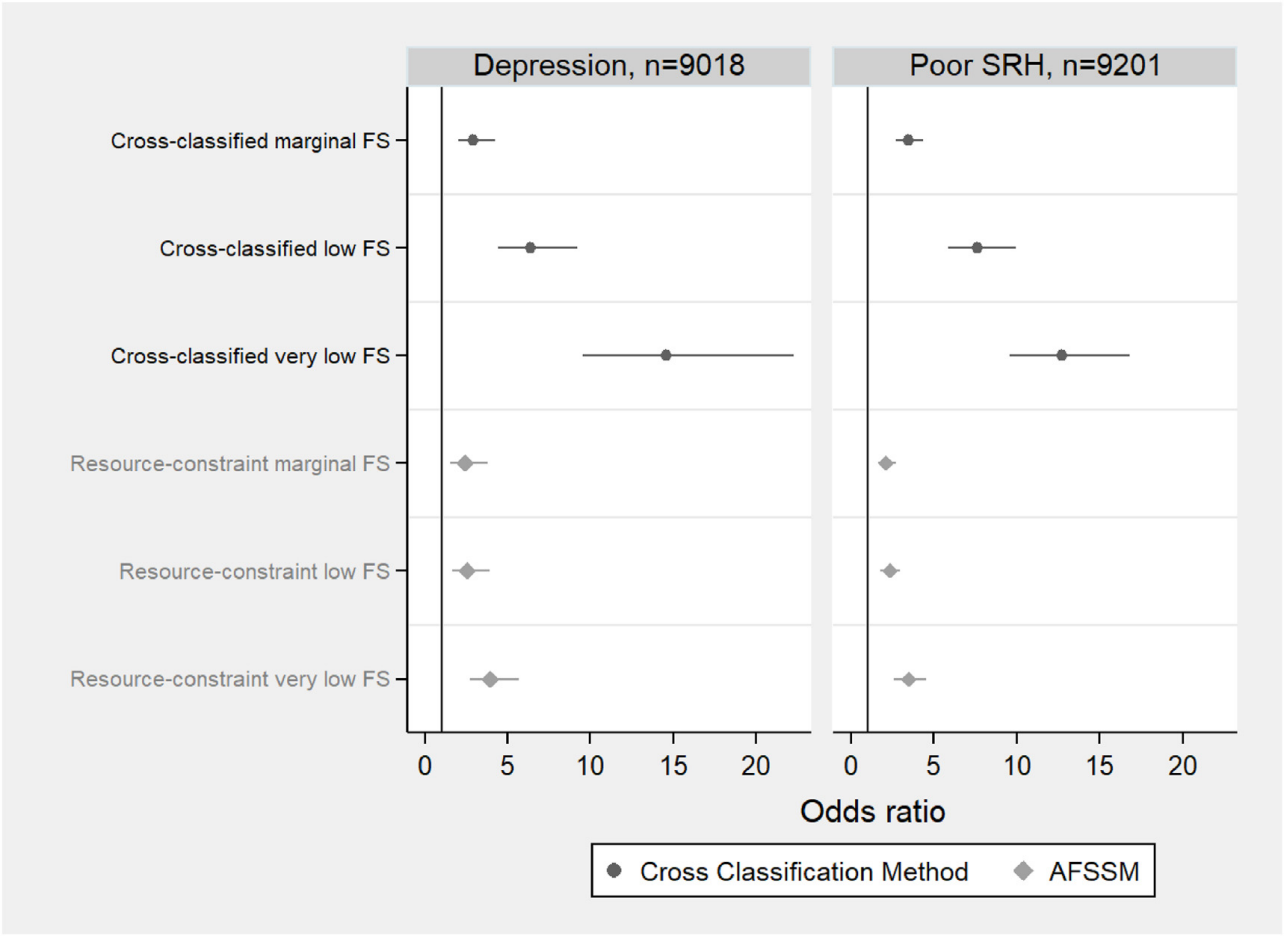
Odds of experiencing depression and poor self-reported health by cross-classification method and standard USDA AFSSM in older adults, NHANES 2007–2018. The cross-classification method refers to the food security status classification according to resource constraints and food-related physical functional limitations. Please refer to Table 1 for a more detailed description of the food security categories based on the cross-classification method. AFSSM refers to the USDA 10-item Adult Food Security Survey Module, which is referred to as “resource-constraint” food security. Poor self-reported health (SRH) is a dichotomous variable and refers to participants who reported fair or poor self-reported health compared with those who reported excellent/very good/good health. Cross-classified high food security based on the proposed cross-classification method and high food security based on the AFSSM measure are the referent categories in both models, adjusted for age, sex, race, marital status, and educational attainment.

Figure 2 showcases the ORs obtained from 4 separate multivariable models. The graph on the left explores the relationships between food security on the basis of the multidimensional cross-classification system and USDA’s food security categories, and depression, which is used as a binary variable (a score of 10 or more indicates presence of depression). Similarly, the graph on the right illustrates the relationship between the various food security measures and poor SRH, and the association is examined using both the cross-classification and USDA’s AFSSM food security methods.

Results demonstrate that cross-classified food insecurity was associated with greater odds of depression and poor SRH, in a dose–response pattern as shown in Figure 2, after adjustment for potential covariates: age, sex, race, marital status and educational attainment. Compared with older adults with cross-classified HFS, participants with cross-classified marginal, low and VLFS had greater odds of having depression, OR = 2.92 [95% confidence interval (CI): 2.01, 4.22], OR = 6.36 (4.40, 9.19), and OR = 14.6 (6.51, 20.8), respectively. Similarly, a robust association was observed between poor SRH and cross-classified food insecurity; OR (95% CI) from the adjusted model were 3.44 (2.74, 4.33), 7.64 (5.88, 9.92), and 12.7 (9.60, 16.8) for older adults with cross-classified marginal, low and VLFS, respectively.

Significant associations were also observed between food security status categories, as determined by the USDA’ AFSSM, and the risk of both depression and poor SRH. In comparison to older adults with HFS, participants with marginal, low and VLFS had higher odds of depression [OR (95% CI): 2.43 (1.55, 3.81), 2.55 (1.66, 3.91), and 3.92 (2.70, 5.68), respectively] as well as higher odds of poor SRH [OR (95% CI): 2.10 (1.63, 2.70), 2.31 (1.80, 2.97), and 3.44 (2.60, 4.56), respectively]. Nevertheless, the ORs exhibit a smaller magnitude in comparison to those obtained from the cross-classified resource-constraint and food-related physical functioning limitations food security models (Figure 2).

A robust inverse association was observed between cross-classified marginal, low, and VLFS and total HEI-2015 scores [regression coefficient (95% CI): –2.11 (–3.19, –1.03), –3.36 (–4.63, –2.08), and –4.74 (–6.79, –2.68), respectively] after accounting for potential confounders. Similarly, we found consistent inverse associations between total HEI-2015 scores and the standard USDA marginal, low, and VLFS statuses. However, no significant differences in the regression coefficients of the models were observed when comparing the cross-classification and standard USDA methods (data not shown).

## Discussion

Older adults with physical functioning limitations may face food insecurity for reasons beyond the economic affordability of food, such as challenges in physically acquiring, preparing, or consuming food. The proposed multidimensional cross-classification method developed in this study enhances the existing USDA AFSSM by extending the food security measure to capture another dimension of food hardship. These dimensions are tied to food-related resource constraints and food-related physical functioning limitations for use among older adults. By incorporating multiple dimensions, this new cross-classification system has the potential to provide a more comprehensive understanding of food insecurity among older adults.

Most existing studies identify food insecurity using the AFSSM scale, which we refer to as resource-constraint food insecurity. Our findings show that a substantial proportion of older adults may have food-related physical conditions that impair their ability, above and beyond resource constraints, to access enough food for a healthy life. Indeed, for the period of 2007–2018, the prevalence of low and VLFS among older adults based on the AFSSM was significantly lower (4.7% and 3.3%, respectively) than that based on the cross-classification method (13.6% and 7.3%, respectively), resulting in differences of 8.9% and 4.0%, respectively. Differences in the prevalence of food insecurity based on these scales suggest that a significant number of older adults are not affirming any of the “resource-constraint” questions, but they are affirming ≥1 of the physical functioning limitations’ questions related to food acquisition, preparation, and consumption, which can also contribute to their food insecurity. Therefore, a screening process for food-related physical functioning limitations could aid in identifying additional older adults who are experiencing food hardship, in particular for reasons not necessarily because of economic constraints. These individuals may be at risk of malnutrition and this measure could potentially be used to help target food assistance interventions and other health services [[36], [37], [38], [39]].

The results also demonstrate that the relationship between the cross-classified low and VLFS, and depression and poor SRH was of higher magnitude than results from the AFSSM alone. The stronger association observed may be because food insecurity due to resource constrains and to physical functioning limitations have been independently linked to depression and poor SRH [8,16,[40], [41], [42]], but not examined jointly until now. Results from this study reinforce the established relationship between food insecurity and both depression and poor SRH observed in prior studies. This also suggests that previous work may be underestimating these associations by considering only 1 dimension of food insecurity at a time. The results also support previous research suggesting that tackling food access and utilization may alleviate some stress, anxiety, and depression [43].

The proposed food security cross-classification yielded similar associations between cross-classified food insecurity and sociodemographic characteristics that have been previously reported in research studies using USDA food security measures. These include race and ethnicity (Black and Hispanic respondents having higher prevalence of cross-classified food insecurity) [[44], [45], [46]], income-to-poverty ratio (those with incomes below 185% of the poverty threshold exhibiting higher cross-classified food insecurity) [46,47], and education level (lower attainment associated with higher cross-classified food insecurity) [46,47]. The number of people in a household and marital status were also among the factors that correlated with the cross-classified scale, as shown in previous studies [46,48]. Notably, individuals who were widowed or separated exhibited a greater risk of cross-classified food insecurity. There was also an association between cross-classified food security and self-reported medical conditions, with cross-classified LFS status being associated with a higher probability of stroke, coronary artery disease, and diabetes, mimicking the same relationship seen between food insecurity and chronic diseases that is reported elsewhere [23,24,[49], [50], [51]].

The primary limitation of this study is the cross-sectional nature of the data, which hinders the ability to imply causal pathways. We also cannot ignore the possibility of other unmeasured confounding factors such as social support and interactions with family and friends, which may moderate the relationship between food insecurity and poor mental health [[52], [53], [54]]. Therefore, future studies examining physical access to food as a subdimension of food insecurity should also evaluate the impact of social support and environmental context [55,56]. We also acknowledge that there are other aspects of physical access to food, such as having a personal vehicle or living close to a supermarket, that can potentially contribute to food insecurity [[57], [58], [59]] However, we concentrated on physical functioning limitations as they are particularly relevant to the older adult population, who are at risk of frailty [60]. Also, both indices of food security are based on self-reports and as such are subject to bias, such as recall bias, social desirability bias, and inaccurate reporting, which may affect the reliability of the results [61].

A critique of our approach may be that the cross-classification method identifies individuals experiencing food insecurity regardless of the reason. However, we chose to construct the food security cross-classification method to more completely reflect the original definition and conceptualization of food security, which occurs when individuals have both physical and economic access to food.

Nevertheless, this study has multiple strengths. A key advantage lies in the proposed cross- classification approach, which integrates 2 distinct aspects related to food access and addresses the challenge of bidimensionality associated with food insecurity. The proposed method also overcomes issues arising from using both scales separately or combining them into a single 16-item tool. It addresses the double counting of food insecurity prevalence by ensuring that participants with both types of food insecurity do not contribute twice to the overall prevalence. Moreover, it appropriately designates 1 severity level for each participant on the basis of the outcomes of both measures, which is not possible with 2 separate scales. This method also provides a more accurate severity level than a single tool, which could mask the true severity because of multiple possibilities corresponding to 1 raw score. This further supports the use of this method to capture food insecurity and its severity for vulnerable populations. Assessing the severity of food insecurity is particularly important as the cross-classified VLFS category had the highest odds of depression and poor SRH and the lowest HEI-2015 total score. This approach has been previously employed to improve the classification of food security in households with children and was found to provide a more accurate depiction of food security in such households, while also reducing biases related to bidimensionality [19]. Finally, using a large sample of older adults, we could demonstrate the consistency of the cross-classified food security method. This method showed strong associations with known correlates of food insecurity and diet quality, as well as expected mental health outcomes [62]. These outcomes were specifically chosen to support and validate the cross-classification method. Importantly, regardless of the underlying food security dimension, assigning food security status using the cross-classification method consistently showed these strong associations.

The objective of this research was to develop a method to estimate food security related to both resource constraints and food-related physical functioning limitations. By doing so, we can identify an additional dimension of food insecurity, and including for the vulnerable older adults who may not otherwise be counted as food insecure. This is especially pertinent when factors such as the ability to shop and prepare meals are not captured by the central dimension of the USDA food security measure. This approach can provide valuable guidance for future studies or programs aiming to integrate both dimensions of food insecurity so that they can effectively evaluate the food security status of older adult populations.

## Author contributions

The authors’ responsibilities were as follows – NRS, AJS: developed the research plan; AJS: analyzed the data and performed statistical analysis; NRS, AJS, AM, ACJ, MPR: wrote the article; and all authors: are responsible for writing and final content, and they have read and approved the final manuscript.

## Funding

This research was supported in part by the USDA Agricultural Research Service Cooperative Agreement (grant #USDA-58-8040-1-006) and the USDA Research, Education and Economics (grant #USDA-58-4000-3-0074). The supporting source only reviewed the manuscript prior to submission but had no involvement or restrictions regarding publication.

## Conflict of interest

The authors report no conflicts of interest. The findings and conclusions in this publication are those of the authors and should not be construed to represent any official USDA or United States Government determination or policy.
