# Supplementary material for: A Method for Assessing the Prevalence of Food Insecurity in Older Adults Based on Resource Constraints and Food-Related Physical Functioning Limitations
Source: Curr Dev Nutr. 2024 Oct 29;8(12):104494. doi: 10.1016/j.cdnut.2024.104494 (PMC11635732; doi:10.1016/j.cdnut.2024.104494)
Supplement: Multimedia component 1 [file mmc1.docx]

**Supplementary Tables**

**Supplementary Table 1.** Frequency of food security categories based on resource constraints and food-related physical functioning limitations among older adults participating in NHANES 2007-2018.

|  | *Resource-constraint food security (AFSSM)* | | | | |
| --- | --- | --- | --- | --- | --- |
| *Food-related physical functioning limitations* | **High food security (HFS)**  (Raw score 0 on AFSSM) | **Marginal food security (MFS)**  (Raw scores 1-2 on AFSSM) | **Low food security (LFS)**  (Raw scores 3-5 on AFSSM) | **Very low food security (VLFS)**  (Raw scores 6-10 on AFSSM) | **Total** |
| **High food-related physical functioning security (H-PFS)**  (Raw score 0 on PFS) | 4285 | 350 | 286 | 171 | 5092 |
| **Marginal food-related physical functioning security (M-PFS)**  (Raw scores 1-2 on PFS) | 2405 | 310 | 259 | 160 | 3134 |
| **Low food-related physical functioning security (L-PFS)**  (Raw scores 3-4 on PFS) | 880 | 201 | 189 | 133 | 1403 |
| **Very low food-related physical functioning security (VL-PFS)**  (Raw scores 5-6 on PFS) | 344 | 86 | 95 | 83 | 608 |
| **Total** | 7914 | 947 | 829 | 547 | 10237 |
